# Sickle Cell Anemia: Variants in the *CYP2D6*, *CAT*, and *SLC14A1* Genes Are Associated With Improved Hydroxyurea Response

**DOI:** 10.3389/fphar.2020.553064

**Published:** 2020-09-09

**Authors:** Sètondji Cocou Modeste Alexandre Yahouédéhou, Joelma Santana dos Santos Neres, Caroline Conceição da Guarda, Suellen Pinheiro Carvalho, Rayra Pereira Santiago, Camylla Vilas Boas Figueiredo, Luciana Magalhães Fiuza, Uche Samuel Ndidi, Rodrigo Mota de Oliveira, Cleverson Alves Fonseca, Valma Maria Lopes Nascimento, Larissa Carneiro Rocha, Corynne Stéphanie Ahouéfa Adanho, Tiago Santos Carvalho da Rocha, Elisângela Vitória Adorno, Marilda Souza Goncalves

**Affiliations:** ^1^Laboratório de Investigação em Genética e Hematologia Translacional, Instituto Gonçalo Moniz, Salvador, Brazil; ^2^Laboratório de Pesquisa em Anemia, Departamento de Análises Clínicas, Faculdade de Farmácia, Universidade Federal da Bahia, Salvador, Brazil; ^3^Department of Biochemistry, Ahmadu Bello University, Zaria, Nigeria; ^4^Ambulatório, Fundação de Hematologia e Hemoterapia da Bahia, Salvador, Brazil

**Keywords:** sickle cell anemia, hydroxyurea, *CYP2D6*, *CYP3A4*, *CAT*, *SLC14A1*, laboratory parameters

## Abstract

Differences in hydroxyurea response in sickle cell anemia may arise due to a series of factors with genetic factors appearing to be predominant. This study aims to investigate the effects of single nucleotide polymorphisms in genes encoding drug-metabolizing enzymes and solute carriers on hydroxyurea response, in patients with sickle cell anemia. For that purpose, a total number of 90 patients with sickle cell anemia were recruited, 45 were undergoing hydroxyurea treatment, while 45 were not under the treatment. Association analyses were performed between *CYP3A4* (rs2740574), *CYP2D6* (rs3892097), *CAT* (rs7943316 and rs1001179), and *SLC14A1* (rs2298720) variants and laboratory parameters. According to our findings, patients with hydroxyurea treatment demonstrated higher HbF levels and a significant improvement in hemolytic, hepatic, inflammatory, and lipid parameters in comparison to those without the treatment. We also found significant associations between the *CYP2D6* (rs3892097), *CAT* (rs7943316 and rs1001179), and *SLC14A1* (rs2298720) variants and an improvement of the therapeutic effects, specifically the hemolytic, hepatic, inflammatory, lipid, and renal parameters. In conclusion, our results highlight the importance of the investigated variants, and their strong association with hydroxyurea efficacy in patients with sickle cell anemia, which may be considered in the future as genetic markers.

## Introduction

Hydroxyurea (HU) is an antineoplastic drug primarily used to treat patients with myeloproliferative syndromes (Bandeira et al., 2004; Kovacic, 2011). In addition to its antimetabolite effects, studies have also demonstrated an anti-sickling action. Accordingly, HU was approved by the U.S. Food and Drug Administration for the treatment of patients with sickle cell anemia (SCA) in the context of a severe clinical profile (King, 2003; Huang et al., 2006; Kolliopoulou et al., 2017). Reports have demonstrated that HU treatment leads to increases in fetal hemoglobin (HbF), mean corpuscular hemoglobin (MCH) and mean corpuscular volume (MCV), as well as decreases in white blood cell (WBC), platelet and reticulocyte counts. Furthermore, HU is also associated with reduced adhesion molecule expression and greater nitric oxide (NO) bioavailability (King, 2003; Rees et al., 2010; Sassi et al., 2010; Silva-Pinto et al., 2013; Yahouédéhou et al., 2019). Clinically, patients with SCA undergoing HU treatment present a lower incidence of painful crises, rates of hospitalization, acute chest syndrome episodes, blood transfusion and mortality (King, 2003; Rees et al., 2010; Sassi et al., 2010; Silva-Pinto et al., 2013). Despite these beneficial effects, studies have also demonstrated inter-individual variations in the therapeutic response to HU (Ware et al., 2002; King, 2003; Ma et al., 2007; Ware, 2010). This may be attributed to therapy adherence, or socioeconomic, environmental, physiological and genetic factors. Nonetheless, genetic factors have been highlighted as one of the most important determinants of variation in HU therapeutic response (Gravia et al., 2014; Yahouédéhou et al., 2018a).

Polymorphisms in genes encoding drug-metabolizing enzymes (DME) and solute carriers may alter the bioavailability of drugs and metabolism, thereby influencing efficiency and toxicity (Sheng et al., 2014). Accordingly, we recently conducted a review of the literature focused on genome-wide association studies that investigated genetic biomarkers effects on HU response and studies that investigated HU metabolism (Yahouédéhou et al., 2018a). Evidence showed the involvement of enzymes of the CYP450 family and catalase in HU metabolism, as well as the association between the urea transporter-B (UTB) and HU response in erythroid cells.

In the present study, two isoenzymes of the CYP450 family (CYP3A4 and CYP2D6) were selected, due to their importance in the metabolism of various endo- or xenobiotics. It was reported that CYP3A4 isoenzyme is more abundant in the liver, can metabolize 50% of commercially available drugs and has substrates such as steroid hormones, analgesics and antihistamines, as well as antitumor and immunosuppressive agents (Božina et al., 2009). The CYP3A4 isoenzyme is encoded by the polymorphic respective gene, located on chromosome 7 (7q22.1) and in particular, the *CYP3A4* -392A>G (rs2740574) is associated with increased gene transcription and CYP3A4 activity (Jin et al., 2005; Božina et al., 2009; Bhatnagar et al., 2010; Maruf et al., 2012; He et al., 2014). As its homolog, CYP2D6 isoenzyme is also more expressed in the liver and encoded by the *CYP2D6* gene located on chromosome 22 (22q13.2). This isoenzyme metabolizes approximately 25% of all known drugs, including antidepressants, antiarrhythmics, analgesics and anticancer agents. Moreover, studies showed the association of the polymorphism *CYP2D6* 1934G>A (rs3892097) with reduced CYP2D6 activity and increased intoxication risk, as well as side effects, following exposure to xenobiotics (Božina et al., 2009; Sayed and Imam, 2012).

In addition to these isoenzymes, we also selected catalase and UTB. Catalase is an important endogen antioxidant enzyme, involved in neutralization pathways of reactive oxygen species. Evidence has shown that it is most abundant in the liver, kidney, and erythrocytes (Forsberg et al., 2001; Babusikova et al., 2013; Liu et al., 2015). Moreover, it was demonstrated that catalase can convert HU into nitrite/nitrate, and HU toxicity *in vivo* is dependent on its activity (Huang et al., 2004; King, 2005; Juul et al., 2010). This enzyme is encoded by the *CAT* gene, located on chromosome 11 (11p13.31) and, according to the latest updates, -21A>T (rs7943316), and -262C>T (rs1001179) variants where are located in the promoter region of this gene, have been associated with reduced transcription and enzyme activity (Perianayagam et al., 2007; Liu et al., 2015). The UTB, also named Solute Carrier Family 14 Member 1 (SLC14A1), is a family of urea transporters important to the regulation of urine concentration (Garcia-Closas et al., 2011; Stewart, 2011; Matsuda et al., 2015; Ebbinghaus et al., 2017). This protein, encoded by the *SLC14A1* gene, located on chromosome 18 (18q12.3), is widely expressed on the plasma membranes of red blood cells and poorly expressed in endothelial cells of the descending rectum vessel in the kidney, brain, ear, testis, intestine, and urinary bladder (Sands, 2002; Garcia-Closas et al., 2011; Rafnar et al., 2011; Ebbinghaus et al., 2017). It is known that several molecules (methylurea, formamide, methylformamide, acetamide, and acrylamide) may transit, rapidly, and passively, *via* channel proteins such as UTB (Esteva-Font et al., 2015; Hou et al., 2017). Interestingly, a study performed in patients with SCA demonstrated an association between the rs12605147 and rs2298720 variants in the *SLC14A1* gene and variations in HU pharmacokinetics (Ware et al., 2011).

Based on this evidence, we conducted the present study, which aimed to investigate, in SCA patients undergoing HU treatment, possible associations between laboratory parameters and the rs2740574, rs3892097, rs7943316, rs1001179, rs2298720 variants, linked to alterations in gene expression and/or enzyme activity.

## Materials and Methods

### Subjects and Ethical Aspects

The present cross-sectional study included patients with SCA (HbSS), all seen at the outpatient clinic of the Hematology and Hemotherapy Foundation of Bahia (HEMOBA), located in Salvador, Brazil. Forty-five (45) patients on HU treatment (HU^+^), as well as 45 who were not using this treatment (HU^-^), were enrolled. Twenty-two (48.89%) HU^+^ patients were female compared to 20 (44.44%) in the HU^-^ group. The median age of the HU^+^ group was 15 years (range: 9–22 years), versus 15.5 years (range: 7–21 years) in the HU^-^ group. All patients reported regular use of folic acid and were in steady-state, defined as the absence of acute crisis and no use of blood transfusion in the three months prior to blood collection. HU dosage ranged between 10.5 and 27.6 mg/kg/day (median: 16.46). The average length of HU treatment was 31.7 months. Patients undergoing chronic transfusion therapy and those with active infection or inflammatory diseases were excluded from the study.

This research protocol received approval from the Institutional Review Board of São Rafael Hospital (protocol number: 1.400.527) and was conducted in compliance with the Declaration of Helsinki (1964) and its revisions. All individuals or their legal guardians provided a signed term of informed consent prior to enrollment in the study.

### Laboratory Parameters

Blood samples were collected by venipuncture in the morning, after 12h of fasting, under standardized conditions. Analyses were performed at the Clinical and Toxicological Analysis Laboratory (LACTFAR) and the Anemia Research Laboratory (LPA), both affiliated with the Pharmaceutical School of the Federal University of Bahia (FACFAR-UFBA), as well as at the Laboratory of Investigation in Genetics and Translational Hematology, Gonçalo Moniz Institute (LIGHT-IGM).

Hematological parameters were evaluated using a Beckman Coulter LH 780 Hematology Analyzer (Beckman Coulter, Brea, California, USA). Qualitative and quantitative profiles of hemoglobin were assessed by high-performance liquid chromatography using an HPLC/Variant II hemoglobin testing system (BIO-RAD, Hercules, CA, USA). Biochemical parameters, as lipid profile, total proteins and fractions, total bilirubin and fractions, lactate dehydrogenase (LDH), alanine aminotransferase (ALT), aspartate aminotransferase (AST) and gamma-glutamyl-transferase (GGT), as well as renal profile and serum iron levels, were assessed using an A25 spectrophotometer autoanalyzer (Biosystems SA, Barcelona, Spain). Alpha-1 antitrypsin (AAT) and C-reactive protein (C-RP) levels were quantified using an Immage 800 system (Beckman Coulter, Fullerton, CA, USA). Serum ferritin was assessed on an Access 2 Immunoassay System (Beckman Coulter, Fullerton, CA, USA).

### Molecular Analysis

Molecular analyses were carried out on genomic DNA obtained from whole blood samples. In overall, *CYP3A4* -392A>G (rs2740574), *CYP2D6* 1934G>A (rs3892097), *CAT* -21A>T (rs7943316), and -262C>T (rs1001179) variants were investigated using the polymerase chain reaction-restriction fragment length polymorphism (PCR-RFLP) technique (Maruf et al., 2012; Sayed and Imam, 2012; Liu et al., 2015). Primers and restriction enzymes used in the PCR-RFLP reactions are presented in **Table S1**. The *SLC14A1* G>A (rs2298720) variant was investigated using the TaqMan method in accordance with the manufacturer’s instructions. We also investigated beta S (β^S^) haplotypes and alpha (α)-thalassemia-2 with the 3.7kb deletion (α^2 del 3.7kb^ thalassemia) since they are associated with alterations in the laboratory parameters of patients with SCA (Camilo-Araújo et al., 2014; Darbari et al., 2014). Beta S haplotypes and α^2 del 3.7kb^ thalassemia were investigated by PCR-RFLP and allele-specific PCR, respectively (Sutton et al., 1989; Chong et al., 2000).

### Statistical Analysis

All statistical analyses were performed using GraphPad Prism 6.0 and SPSS 17.0, with *p*<0.05 considered statistically significant. The Shapiro-Wilk test was used to determine quantitative variable distributions. Mean values between two groups were compared using the unpaired t-test for variables with a normal distribution, while the Mann-Whitney *U* test was used for variables with non-normal distributions. ANOVA or Kruskal Wallis were used to compare mean values between more than two groups according to distribution. Frequencies of qualitative variables were also calculated. The Chi-square test (χ2-test) with Yates correction and Fischer’s Exact test were used to investigate differences in genotypic and allelic frequencies between the two groups. Associations between parameters and polymorphisms were evaluated using codominant (wild type vs heterozygote vs variant), dominant (wild type vs heterozygote/variant), and recessive genetic (wild type/heterozygote vs variant) models, and multivariate linear regression analysis was employed to investigate the influence of the investigated variants on laboratory parameters. Results were expressed as mean ± standard deviation (SD), median (minimum-maximum), or number or frequency where appropriate.

## Results

### Laboratory Parameters of Patients With or Without HU Treatment

The hematological and biochemical parameters of patients receiving, or not, HU treatment are presented in **Table S2**. HU treatment was associated with an increase in HbF levels and, consequently, a reduction in HbS levels (*p*<0.05). The analysis of biomarkers related to hemolysis and hepatic injury demonstrated an association between HU treatment and increases in hemoglobin, hematocrit, MCV and MCH, as well as reductions in reticulocytes, mean corpuscular hemoglobin concentration (MCHC), red blood cell distribution width (RDW), total bilirubin, AST, and LDH (*p*<0.05). Regarding leukocyte and platelet profiles, decreased counts of WBC, neutrophils, eosinophils, lymphocytes, monocytes, platelets, as well as reduction in plateletcrit levels (*p*<0.05) were seen in patients undergoing HU treatment compared to those who were not. HU was also significantly associated with increased high-density lipoprotein cholesterol (HDL-C) levels.

### Frequencies of Investigated Polymorphisms in Patients With or Without HU Treatment

The analysis of genotypic and allelic distributions of the investigated polymorphisms revealed a lower frequency of the *CYP2D6* 1934GA+AA genotype in patients undergoing HU treatment compared to those who were not (*p* = 0.0149). Moreover, lower and higher frequencies of the *CYP3A4* -392G (*p* = 0.0248) and *CAT* -21T (*p* = 0.0485) variant alleles were respectively observed in patients on HU compared to those who did not receive this treatment (**Table S3**). The rs7943316, rs1001179 and rs2298720 variants were found to be in Hardy-Weinberg Equilibrium (HWE), while rs3892097 and rs2740574 variants were not. The genotypic distribution of β^S^ haplotypes and α-thalassemia according to HU treatment are presented in **Table S4**; no significant differences were observed between the two groups (*p*>0.05).

### Associations Between Polymorphisms and Laboratory Parameters

**Figure 1:**
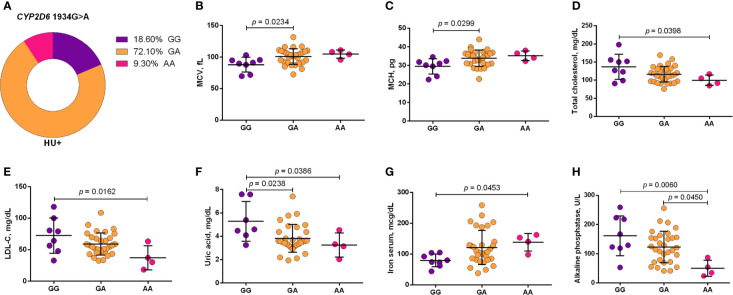
Frequency of *CYP2D6* 1934G>A in patients with sickle cell anemia (SCA) undergoing hydroxyurea (HU) therapy **(A)** and its association with laboratory parameters using the co-dominant genetic model **(B–H)**. The co-dominant genetic model compared three genotype groups (wild type *vs* heterozygote *vs* variant). MCV, mean corpuscular volume; MCH, mean corpuscular hemoglobin; LDL-C, low-density lipoprotein cholesterol; ALT, alanine aminotransferase; ANOVA or Kruskal-Wallis where appropriate.

**Figure 2:**
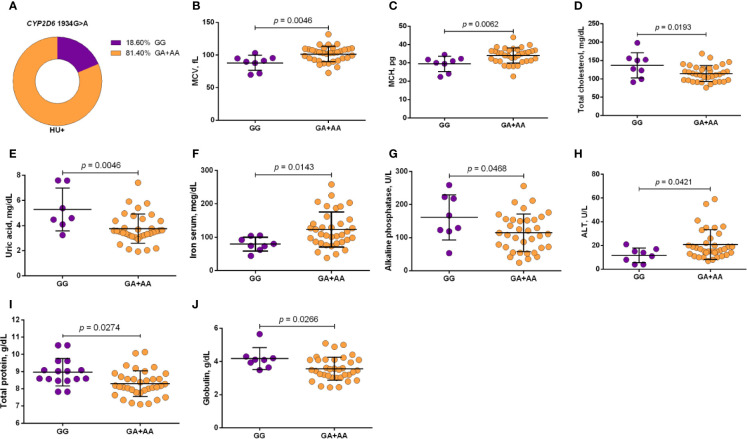
Frequency of *CYP2D6* 1934G>A in patients with sickle cell anemia (SCA) undergoing hydroxyurea (HU) therapy **(A)** and its association with laboratory parameters using the dominant genetic model **(B–J)**. The dominant genetic model compared two genotype groups (wild type *vs* heterozygote/variant). MCV, mean corpuscular volume; MCH, mean corpuscular hemoglobin; ALT, alanine aminotransferase; unpaired t-test or Mann Whitney *U* test where appropriate.

**Figure 3:**
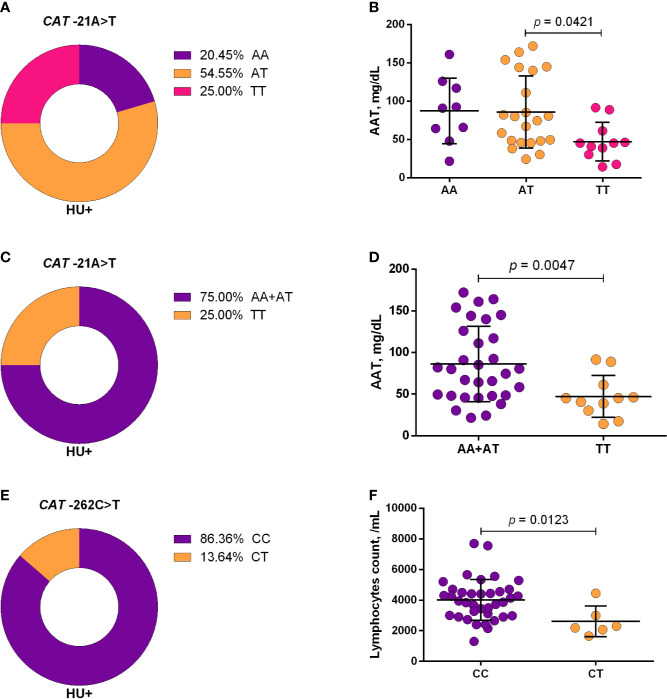
Frequencies of *CAT* -21A>T **(A, C)** and -262C>T **(E)** and respective associations with laboratory parameters, using co-dominant **(B, F)**, and recessive **(D)** models. The co-dominant genetic model compared three genotype groups (wild type *vs* heterozygote *vs* variant), while the recessive genetic model compared two genotype groups (wild type/heterozygote *vs* variant). AAT, alpha 1-antitrypsin; ANOVA, Kruskal-Wallis; unpaired t-test or Mann-Whitney *U* test where appropriate.

**Figure 4:**
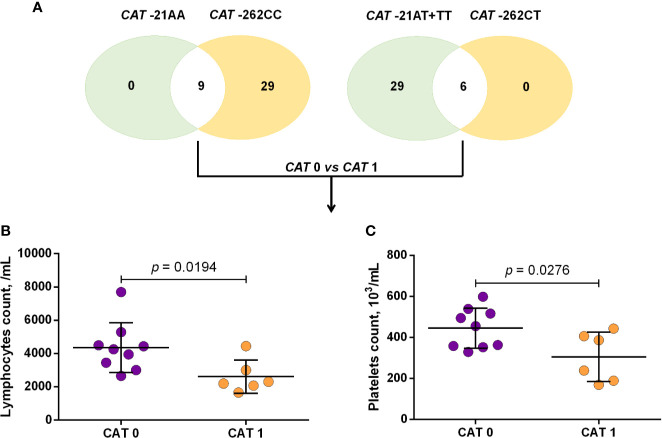
Number of sickle cell anemia (SCA) patients receiving hydroxyurea (HU) who carry either two wild type or two variant alleles in both the *CAT* -21A>T and -262C>T variants **(A)** and association of the *CAT* haplotype with laboratory parameters **(B, C)**. *CAT* 0: patients with both *CAT* -21AA and -262CC genotypes, *CAT* 1: patients with both *CAT* -21AA+TT and -262CT genotypes, unpaired t-test or Mann Whitney *U* test where appropriate.

**Figure 5:**
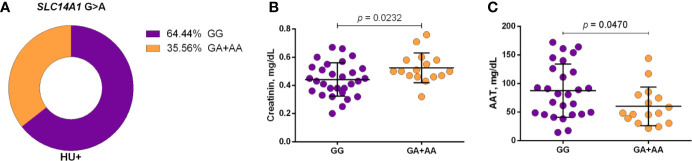
Frequency of *SLC14A1* G>A (rs2298720) in patients with sickle cell anemia (SCA) undergoing hydroxyurea (HU) therapy **(A)** and its association with laboratory parameters under dominant genetic model analysis **(B, C)**. Two genotype groups (wild type *vs* heterozygote/variant) were compared. AAT, alpha 1-antitrypsin; unpaired t-test or Mann Whitney *U* test where appropriate.

Using a codominant genetic model, HU^+^ patients who had the *CYP2D6* heterozygote (1934GA) or variant (1934AA) genotypes presented significantly increased MCV, MCH and iron serum, as well as reduced total cholesterol, low-density lipoprotein cholesterol (LDL-C), uric acid, and alkaline phosphatase (ALP) compared with carriers of the wild type (1934GG) genotype. Furthermore, SCA HU^+^ patients who were carriers of the heterozygote genotype, *CYP2D6* 1934GA, presented intermediary values in laboratory investigations compared with carriers of the *CYP2D6* wild type (1934GG) and variant (1934AA) genotypes (**Figure 1**). Using a dominant genetic model for analysis in HU^+^ patients, associations between *CYP2D6* 1934G>A and significantly increased MCV, MCH, iron serum, and ALT were observed, in addition to significant decreases in total cholesterol, uric acid, ALP, total protein and globulin (**Figure 2**). *CAT* -21A>T demonstrated a significant association with reduced AAT concentrations under both co-dominant and recessive genetic models, while *CAT* -262C>T was found to be significantly associated with reduced lymphocyte counts in HU^+^ patients (**Figure 3**). HU^+^ patients who were carriers of heterozygote and variant genotypes of both *CAT* -21A>T and -262C>T presented reductions in lymphocyte and platelet counts compared to carriers of the wild type genotypes of both polymorphisms (**Figure 4**). Finally, the *SLC14A1 G>A* (rs2298720) was found to be significantly associated with elevated creatinine and reduced AAT in HU^+^ patients (**Figure 5**). None of these associations was detected in patients who did not receive HU. Moreover, *CYP3A4* -392A>G was not found to be clinically significant with respect to the parameters investigated.

### Multivariate Linear Regression Analyses

Based on the findings of our association analyses among HU^+^ patients, multivariate linear regression analyses were conducted, including β^S^ haplotypes and α-thalassemia, as well as some laboratory parameters as confounding variables. Our analysis using the dominant genetic model revealed independent associations between the *CYP2D6* 1934G>A variant and increases in MCV, MCH, and iron serum levels, as well as decreases in total cholesterol, uric acid, total protein and globulin levels. We also observed an independent association between the *CAT* -21A>T variant and AAT using the recessive genetic model. All statistically significant results from our regression analyses are presented in **Table 1**.

**Table 1:** Multivariate linear regression models of *CYP2D6* 1934G>A and *CAT* -21A>T and -262C>T variants in patients with sickle cell anemia (SCA) undergoing hydroxyurea (HU) treatment.

Dependent variables	Independent variables	β	R	*p*1	*p*2
*CYP2D6* 1934G>A					
MCV, fL	CYP2D6*	0.42	0.54	**0.006**	**0.008**
	HAP	0.09		0.544	
	Tal	-0.34		**0.031**	
MCH, pg	CYP2D6*	0.41	0.57	**0.006**	**0.004**
	HAP	0.13		0.364	
	Tal	-0.40		**0.010**	
Total cholesterol, mg/dL	CYP2D6*	-0.33	0.53	**0.028**	**0.008**
	HAP	-0.04		0.788	
	Tal	0.42		**0.008**	
Uric acid, mg/dL	CYP2D6*	-0.41	0.43	**0.007**	**0.018**
	HAP	-0.10		0.480	
Iron serum, mcg/dL	CYP2D6*	0.33	0.39	**0.030**	**0.035**
	HAP	0.20		0.170	
Total protein, g/dL	CYP2D6*	-0.32	0.44	**0.032**	**0.013**
	HAP	-0.30		**0.044**	
Globulin, g/dL	CYP2D6*	-0.31	0.54	**0.030**	**0.004**
	HAP	-0.38		**0.009**	
	Hemoglobin	-0.26		0.066	
*CAT* -21A>T					
AAT, mg/dL	CAT**	-0.39	0.40	**0.011**	**0.035**
	HAPCAT	0.51		0.731	
*CAT* -262C>T					
Lymphocytes, /mL	CAT*	-0.22	0,51	0.144	**0.014**
	HAP	0.34		**0.028**	
	Tal	-0.35		**0.026**	

MCV, mean corpuscular volume; MCH, mean corpuscular hemoglobin; HAP, β^S^ haplotype; Tal, α^2 del 3.7kb^ thalassemia; R, coefficient of determination; β, coefficient of regression; p1, p value of the independent variable; p2, p value of the model; *, dominant genetic model; **, recessive genetic model.

## Discussion

The present study investigated the possible influence of variants in genes encoding DME and solute carrier on SCA patients’ response to HU treatment. As expected, in contrast to SCA patients who did not receive HU, those who received this treatment presented increased HbF levels and improvements in hemolytic, hepatic and inflammatory profiles (hemoglobin, hematocrit, MCV, MCH, reticulocytes, MCHC, RDW, AST, total bilirubin, LDH, WBC, neutrophils, eosinophils, lymphocytes, monocytes, platelets, and plateletcrit). These findings corroborate previous studies, which also demonstrated improvements in hemolytic, hepatic and inflammatory profiles in patients with SCA undergoing HU therapy (de Souza Santos and Maia, 2010; Voskaridou et al., 2010; de Souza Torres et al., 2012; Pallis et al., 2014; Belini Junior et al., 2015; Shome et al., 2016; Quarmyne et al., 2017; Colombatti et al., 2018). We further observed an increase in HDL-C concentration, demonstrating the effect of HU on lipid metabolism, which is consistent with our previous results (Yahouédéhou et al., 2018b; Yahouédéhou et al., 2019).

The present analysis of genotype and/or allele distribution of rs2740574, rs3892097, and rs7943316 revealed frequencies divergent to those observed in other studies conducted in different patient populations, as well as healthy controls (Maruf et al., 2012; Sayed and Imam, 2012; Liu et al., 2015). Contrarily, the frequencies of the rs1001179 and rs2298720 observed herein corroborate previously published results (Angona et al., 2013; Liu et al., 2015). Furthermore, we found a significantly reduced frequency of the *CYP2D6* 1934GA+AA genotype in patients receiving HU compared to those who did not, as well as significantly lower and higher frequencies of the *CYP3A4* -392G and *CAT* -21T allelic variants, respectively, in patients on HU versus those who did not receive this treatment.

Association analyses of polymorphisms with laboratory parameters revealed interesting results. SCA patients who were carriers of the variant (AA) genotype presented more pronounced alterations in response to HU treatment compared to those with the wild type (GG) genotype, indicating an association between the *CYP2D6* 1934G>A and an improvement in HU effects, that might be correlated with the number of variant A allele carried. Indeed, patients with the heterozygote (GA) genotype presented intermediary values on laboratory parameters compared to those with the wild type (GG) or homozygote variant (AA) genotypes. Reports have shown that the variant A allele leads to the incorrect splicing of mRNA, the formation of trunked protein, the reduction in enzymatic activity and a poor metabolism in patients with the variant (AA) genotype (Sayed and Imam, 2012). Hence, our findings suggest that this isoenzyme may inactivate HU or accelerate its elimination, which could explain the lessened effects in response to HU seen in rapid metabolizers (i.e., carriers of wild type GG genotype). Furthermore, in comparison to carriers of the wild type (GG) genotype, we observed that 95.65% of patients who received HU and were carriers of the heterozygote (GA) or variant (AA) genotypes presented MCV>98fL. This further reinforces the association between the variant A allele and improvements in HU effects, as report showed positive correlation between MCV and HbF levels and, consequently, improved laboratory and clinical profiles (Borba et al., 2003).

Studies have demonstrated that HU is metabolized by catalase and that the rs7943316 and rs1001179 variants are associated with a reduction in or the absence of enzyme production (Huang et al., 2004; Juul et al., 2010; Liu et al., 2015). The present study observed associations between the *CAT*-21TT and *CAT*-262CT genotypes in patients receiving HU with respect to reductions in inflammatory biomarkers, such as AAT levels and lymphocyte counts. In addition, carriers of heterozygote and variant genotypes of both *CAT* -21A>T and -262C>T also exhibited significant reductions in lymphocyte and platelet counts compared to carriers of the wild type genotypes of both polymorphisms. These reductions in both lymphocytes and AAT, specifically in those who were carriers of variant alleles, may be due to the positive correlation, which exists between these inflammatory biomarkers in individuals undergoing HU therapy. Moreover, studies have demonstrated that levels of AAT, known as an acute-phase protein with anti-inflammatory properties, can decrease during HU therapy, either as a consequence or a cause of reductions in WBC counts (Pallis et al., 2014; Yahouédéhou et al., 2019). Hence, our findings suggest the likelihood that lower catalase expression and activity may lead to higher HU bioavailability, which could explain the associations seen herein between the *CAT* -21A>T and -262C>T and improvement in inflammatory biomarkers.

Regarding the UTB, a recent study demonstrated a correlation between the upregulation of *SCL14A1* and higher *HBG2* expression in erythroid cells treated with HU (Walker and Ofori-Acquah, 2017). Another study involving patients with SCA reported an association between the polymorphism rs2298720 and alterations in HU pharmacokinetic parameters (Ware et al., 2011). In the present study, we found an association between the variant A allele of the polymorphism rs2298720 and elevated creatinine and reduced AAT concentrations in patients undergoing HU treatment. This results corroborate our previous findings, which observed a negative correlation between AAT and creatinine in patients with sickle cell disease, i.e., with HbSS, HbSC, or HbSβ^+^ (Carvalho et al., 2017). Moreover, it was reported that the accumulation of urea, an HU analog, might occur in human urothelial cells due to low *SLC14A1* expression (Hou et al., 2017). Considering these findings together, it is possible to speculate that the polymorphism rs2298720 may also be associated with diminished HU elimination, resulting in increased bioavailability. Moreover, as a result of reduced AAT concentrations, HU treatment may lead to an increase in creatinine serum levels, suggesting the beneficial effect of the variant A allele on inflammatory and renal dysfunction biomarkers.

The results of the multivariate regression analyses, which were performed using β^S^ haplotypes and α-thalassemia, as well as some laboratory biomarkers as confounding variables, confirmed the independent association of *CYP2D6* 1934G>A, *CAT* -21A>T, and -262C>T with several of these parameters. Accordingly, it will be interesting to investigate, in patients receiving HU who are carriers of these variant alleles, the clinical repercussions of the presently observed alterations in laboratory parameters.

## Conclusion

The present study investigated the effects of specific variants in genes encoding DME and solute carriers on SCA patients’ response to HU treatment. Our results indicate that the *CYP2D6* (rs3892097), *CAT* (rs7943316 and rs1001179), and *SLC14A1* (rs2298720) variants are associated with HU efficacy, based on the laboratory parameters performed. Accordingly, we suggest that these variants are linked to the reduced metabolism or elimination of HU, which may increase its therapeutic effects in patients with SCA. Future investigation of this hypothesis might be the stepping-stone for a better understanding of the HU metabolic pathway and to consider these variants as genetic markers of HU response.

## Data Availability Statement

All datasets presented in this study are included in the article/**Supplementary Material**.

## Ethics Statement

The studies involving human participants were reviewed and approved by Institutional Review Board of the São Rafael Hospital. Written informed consent to participate in this study was provided by the participants’ legal guardian/next of kin.

## Author Contributions

SY, EA, and MG conceived and designed the experiments. SY, JN, CG, SC, RS, CVBF, LF, UN, and RO collected the samples. SY, JN, CG, SC, RS, CVBF, LF, CAF, CA, and TR performed the experiments. SY and JN analyzed the data. VN and LR followed the patients. SY drafted the manuscript. JN and CG contributed to the writing of the manuscript. EA and MG supervised the study and critically revised the manuscript. All authors contributed to the article and approved the submitted version.

## Funding

This work was supported by grants from the Conselho Nacional de Desenvolvimento Científico e Tecnológico (CNPq) (470959/2014- 2 and 405595/2016-6) (MG), Programa Inova Fiocruz - Edital Geração de Conhecimento (VPPCB-007-FIO-18-2-66) (MG) and the Coordenação de Aperfeiçoamento de Pessoal de Nível Superior - Brasil (CAPES) - Finance Code 001 (SY, SC, and RS). The sponsors of this study are public or nonprofit organizations that support science in general.

## Conflict of Interest

The authors declare that our research was conducted in the absence of any commercial or financial relationships that could be construed as a potential conflict of interest.
