# Supplementary material for: Sickle Cell Anemia: Variants in the CYP2D6, CAT, and SLC14A1 Genes Are Associated With Improved Hydroxyurea Response
Source: Front Pharmacol. 2020 Sep 9;11:553064. doi: 10.3389/fphar.2020.553064 (PMC7510454; doi:10.3389/fphar.2020.553064)
Supplement: Supplementary file 1 [file DataSheet_1.docx]

Supplementary Material

**Table S1**: Primers and restriction enzymes used in PCR-RFLP reactions to investigate selected polymorphisms

| **Polymorphism** | **Method** | **Primer** | **Restriction enzyme** | **Reference** |
| --- | --- | --- | --- | --- |
| *CYP3A4* -392A>G  (rs2740574) | PCR-RFLP | F: 5’- GGA ATG AGG ACA GCC ATA GAG ACA AGG GGA -3’  R: 5’- CCT TTC AGC TCT GTG TTG CTC TTT GCT G -3’ | MboII | Maruf et al., 2012 |
| *CYP2D6* 1934G>A  (rs3892097) | PCR-RFLP | F: 5’- GCC TTC GCC AAC CAC TCC G -3’  R: 3’- AAA TCC TGC TCT TCC GAG GC -3’ | MvaI | Sayed and Imam, 2012 |
| *CAT* -21A>T  (rs7943316) | PCR-RFLP | F: 5’- AAT CAG AAG GCA GTC CTC CC -3’  R: 5’- TCG GGG AGC ACA GAG TGT AC -3’ | HinfI | Liu et al., 2015 |
| *CAT* -262C>T  (rs1001179) | PCR-RFLP | F: 5’- AGA GCC TCG CCC CGC CGG ACC G -3’  R: 5’- TAA GAG CTG AGA AAG CAT AGC T -3’ | SmaI | Liu et al., 2015 |

**Table S2**: Laboratory parameters of patients with SCA receiving or not HU therapy

| **Parameter** | **HU^+^ (N=45)**  **Mean ± SD** | **HU^-^ (N=45)**  **Mean ± SD** | ***p*** |
| --- | --- | --- | --- |
| **Hemoglobin** |  |  |  |
| HbF, % | 11.90±5.70 | 7.05±4.67 | **<0.0001*** |
| HbS, % | 85.05±5.64 | 89.72±5.20 | **0.0001*** |
| **Hemolysis** |  |  |  |
| RBC, x10^6^/mL | 2.75±0.52 | 2.71±0.52 | 0.7159* |
| Hemoglobin, g/dL | 8.93±1.12 | 7.95±0.82 | **<0.0001**** |
| Hematocrit, % | 26.74±3.57 | 23.32±2.87 | **<0.0001*** |
| Reticulocyte, % | 124963±67866 | 153473±62318 | **0.0419*** |
| MCV, fL | 98.60±12.65 | 86.49±9.61 | **<0.0001*** |
| MCH, pg | 33.07±4.45 | 29.89±3.65 | **0.0004*** |
| MCHC, % | 33.52±0.86 | 34.55±1.10 | **<0.0001*** |
| RDW, % | 20.77±3.62 | 24.56±3.31 | **<0.0001*** |
| Erythroblast (/10^2^ leukocytes) | 1.59±5.04 | 1.33±2.66 | 0.4384** |
| **Leukocytes** |  |  |  |
| WBC, /mL | 9694±3069 | 12393±2162 | **<0.0001**** |
| Neutrophil, /mL | 4511±2048 | 5811±2009 | **0.0033*** |
| Eosinophil, /mL | 367±358 | 612±427 | **0.0044*** |
| Lymphocyte, /mL | 3808±1365 | 4460±1319 | **0.0243*** |
| Monocyte, mL | 843±470 | 1325±586 | **<0.0001*** |
| **Platelets** |  |  |  |
| Platelet, x10^3^/mL | 388±152 | 443±86 | **0.0110**** |
| Plateletcrit, % | 0.29±0.12 | 0.35±0.07 | **0.0005**** |
| MPV, fL | 7.88±0.87 | 8.08±0.82 | 0.2519* |
| PDW, % | 16.37±0.75 | 16.18±0.56 | 0.2181* |
| **Hemolytic plus hepatic** |  |  |  |
| Total bilirubin, mg/dL | 2.65±1.77 | 3.36±1.55 | **0.0454*** |
| Direct bilirubin, mg/dL | 0.38±0.15 | 0.43±0.18 | 0.1532* |
| Indirect bilirubin, mg/dL | 2.26±1.76 | 2.92±1.51 | 0.0597* |
| Lactate dehydrogenase, U/L | 991.58±344.18 | 1289.35±420.10 | **0.0004*** |
| Iron serum, mcg/dL | 113.29±50.81 | 100.62±50.51 | 0.2388* |
| Aspartate aminotransferase, U/L | 41.31±16.53 | 52.78±18.09 | **0.0023*** |

**Table S2**: Continued

| **Lipids and glucose** |  |  |  |
| --- | --- | --- | --- |
| Total cholesterol, mg/dL | 118.44±25.26 | 123.35±26.20 | 0.3678* |
| HDL-C, mg/dL | 38.00±8.45 | 33.60±8.14 | **0.0137*** |
| LDL-C, mg/dL | 59.74±21.06 | 66.14±22.05 | 0.1630* |
| Triglycerides, mg/dL | 99.48±43.45 | 117.87±54.12 | 0.0791* |
| Glucose, mg/dL | 83.82±9.30 | 82.22±7.37 | 0.3681* |
| **Renal** |  |  |  |
| Urea, mg/dL | 18.69±7.99 | 16.26±4.42 | 0.3229** |
| Creatinine, mg/dL | 0.47±0.12 | 0.43±0.15 | 0.1486* |
| **Hepatic** |  |  |  |
| Alanine aminotransferase, U/L | 19.07±11.83 | 21.24±10.71 | 0.3624* |
| γ-glutamine aminotransferase, U/L | 23.91±15.88 | 26.13±19.02 | 0.5490* |
| Total protein, g/dL | 8.44±0.79 | 8.29±0.84 | 0.3759* |
| Albumin, g/dL | 4.76±0.33 | 4.79±0.34 | 0.6893* |
| Globulin, g/dL | 3.68±0.71 | 3.51±0.65 | 0.2294* |
| Alkaline phosphatase, U/L | 124.42±61.01 | 133.38±71.83 | 0.5255* |
| **Inflammatory** |  |  |  |
| Uric acid, mg/dL | 4.02±1.35 | 3.95±1.08 | 0.8053* |
| Ferritin, ng/dL | 407.20±345.49 | 243.10±364.39 | 0.0960* |
| C-reactive protein, mg/L | 6.34±9.45 | 4.54±3.41 | 0.5328** |
| Alpha 1 antitrypsin, mg/dL | 77.09±44.11 | 75.65±43.89 | 0.8806* |

RBC: red blood cell, MCH: mean corpuscular hemoglobin, MCV: mean corpuscular volume, MCHC: mean corpuscular hemoglobin concentration, HbS: variant S hemoglobin, HbF: Fetal hemoglobin, RDW: red cell distribution width, HDL-C: high-density lipoprotein cholesterol, LDL-C: low-density lipoprotein cholesterol, WBC: white blood cell, MPV: mean platelet volume, PDW: platelet distribution width, N: number of individual, SD: standard deviation, * Unpaired T test, ** Mann Whitney *U* test

**Table S3**: Genotypic and allelic frequencies of polymorphisms in patients with SCA receiving or not HU therapy

| **SNP** |  | **Genotype, N (%)** | | |  |  | **Allele, N1 (%)** | | |  |
| --- | --- | --- | --- | --- | --- | --- | --- | --- | --- | --- |
|  |  | Total | HU^+^ | HU^-^ | *p*^#^ |  | Total | HU^+^ | HU^-^ | *p* |
| ***CYP2D6* 1934G>A** | GG | 09 (10.34) | 08 (18.60) | 01 (2.27) | **0.0149** | G | 88 (50.57) | 47 (54.65) | 41 (46.59) | 0.3619 |
| **(rs3892097)** | GA | 70 (80.46) | 31 (72.10) | 39 (88.64) |  | A | 86 (49.43) | 39 (45.35) | 47 (53.41) |  |
|  | AA | 8 (9.20) | 04 (9.30) | 04 (9.09) |  |  |  |  |  |  |
|  | GA+AA | 78 (89.66) | 35 (81.39) | 43 (97.73) |  |  |  |  |  |  |
| ***CYP3A4* -392A>G** | AA | 32 (36.36) | 20 (44.44) | 12 (27.91) | 0.1644 | A | 96 (54.54) | 57 (63.33) | 39 (45.35) | **0.0248** |
| **(rs2740574)** | AG | 32 (36.36) | 17 (37.78) | 15 (34.88) |  | G | 80 (45.46) | 33 (36.67) | 47 (54.65) |  |
|  | GG | 24 (27.27) | 08 (17.78) | 16 (37.21) |  |  |  |  |  |  |
|  | AG+GG | 56 (63.64) | 25 (55.55) | 31 (72.09) |  |  |  |  |  |  |
| ***CAT* -21A>T^*^** | AA | 26 (29.55) | 09 (20.45) | 17 (38.64) | 0.1019 | A | 98 (55.68) | 42 (47.73) | 56 (63.64) | **0.0485** |
| **(rs7943316)** | AT | 46 (52.27) | 24 (54.55) | 22 (50.00) |  | T | 78 (44.32) | 46 (52.27) | 32 (36.36) |  |
|  | TT | 16 (18.18) | 11 (25.00) | 05 (11.36) |  |  |  |  |  |  |
|  | AT+TT | 62 (70.45) | 35 (79.54) | 27 (61.36) |  |  |  |  |  |  |
| ***CAT* -262C>T^*^** | CC | 78 (87.64) | 38 (86.36) | 40 (88.89) | 0.9682 | C | 167 (93.82) | 82 (93.18) | 85 (94.44) | 0.9693 |
| **(rs1001179)** | CT | 11 (12.36) | 06 (13.64) | 05 (11.11) |  | T | 11 (6.18) | 6 (6.82) | 5 (5.56) |  |
|  | TT | 0 (0) | 0 (0) | 0 (0) |  |  |  |  |  |  |
|  | CT+TT | 11 (12.36) | 06 (13.64) | 05 (11.11) |  |  |  |  |  |  |
| ***SLC14A1* G>A^*^** | GG | 64 (71.11) | 29 (64.45) | 35 (77.78) | 0.2449 | G | 153 (85.0) | 73 (81.11) | 80 (88.89) | 0.2104 |
| **(rs2298720)** | GA | 25 (27.78) | 15 (33.33) | 10 (22.22) |  | A | 27 (15.0) | 17 (18.89) | 10 (11.11) |  |
|  | AA | 1 (1.11) | 1(2.22) | 0 (0) |  |  |  |  |  |  |
|  | GA+AA | 26 (28.89) | 16 (35.55) | 10 (22.22) |  |  |  |  |  |  |

HU: hydroxyurea, N: number of individual, N1: number of chromosomes, * the polymorphism was in Hardy-Weinberg Equilibrium, ^#^ *p* value refers to comparisons between wild type genotype *vs* heterozygote/variant genotypes, χ2-test with Yates correction and Fischer’s Exact test performed where appropriate

**Table S4**: Genotypic frequencies of β^S^ haplotype and α-thalassemia in SCA patients receiving or not HU therapy

|  | **Patients with SCA** | |  |
| --- | --- | --- | --- |
|  | **HU^+^, N (%)** | **HU^-^, N (%)** | ***p* value** |
| **β^S^ haplotype** |  |  |  |
| BEN/BEN | 15 (33.33%) | 21 (46.67%) | 0.282 |
| CAR/BEN and CAR/CAR | 30 (66.67%) | 24 (53.33%) |  |
| **α^2del 3.7kb^ thalassemia** |  |  |  |
| αα/αα | 30 (75%) | 30 (75%) | 1.000 |
| -α/αα and -α/-α | 10 (25%) | 10 (25%) |  |

SCA: sickle cell anemia, HU: hydroxyurea, BEN: Benin haplotype, CAR: Central African Republic haplotype, %: frequency, N: number, χ2-test with Yates correction was performed
